# A Treatise on Dentistry

**Published:** 1853-07

**Authors:** 


*A Treatise on Dentistry.* By Samuel Fowell, Dentist. London. Simpkins, Marshall & Co., 1853.

This is a very neat popular treatise on the teeth and the method of preserving them from the inroads of disease. It is issued for the instruction of patients, and the author has done wisely in this matter, for true science has nothing to lose and every thing to gain by the extension of knowledge in reference to its principles among the people. It is only quackery that lives by mystery. Take away from it that element and it perishes by inanition.

